# Patient-Related Factors Associated with Adherence to Recommendations Made by a Fracture Liaison Service: A Mixed-Method Prospective Study

**DOI:** 10.3390/ijerph15050944

**Published:** 2018-05-09

**Authors:** Mireille Luc, Hélène Corriveau, Gilles Boire, Johanne Filiatrault, Marie-Claude Beaulieu, Isabelle Gaboury

**Affiliations:** 1 Department of Family Medicine and Emergency Medicine, Université de Sherbrooke, Sherbrooke, QC J1H 5N4, Canada; Mireille.Luc@USherbrooke.ca (M.L.); Marie-Claude.Beaulieu@USherbrooke.ca (M.-C.B.); 2 School of Rehabilitation, Université de Sherbrooke, Sherbrooke, QC J1H 5N4, Canada; Helene.Corriveau@USherbrooke.ca; 3 Division of Rheumatology, Department of Medicine, Université de Sherbrooke, Sherbrooke, QC J1H 5N4, Canada; Gilles.Boire@USherbrooke.ca; 4 School of Rehabilitation, Université de Montréal, Montreal, QC H3C 3J7, Canada; Johanne.Filiatrault@UMontreal.ca

**Keywords:** fragility fracture, Fracture Liaison Service, adherence, health behaviors

## Abstract

A Fracture Liaison Service (FLS) has been calculated to be a cost-effective model of care for patients with fragility fracture (FF). Cost-effectiveness can be achieved when adherence to bone health recommendations from FLS staff is high. This prospective study combined participants’ telephone longitudinal survey data (intervention group, *n* = 354) and interviews with 16 individuals from FLS in three health regions of the province of Quebec (Canada). Participants were recruited between January 2013 and April 2015. Regression models were fit to examine the relationship between participant-related factors and adherence at 12 months to osteoporosis medication, vitamin D supplementation, and participation in physical activity. Participants acknowledging FF as a consequence of osteoporosis were more likely to adhere to medication (odds ratio (OR) 2.5; *p* = 0.001) and vitamin D supplementation (OR 2.3; *p* = 0.01). Paradoxically, the same participants were less prone to engage in physical activity (OR 0.5, *p* = 0.01). Qualitative interviews suggested that feedback from FLS coordinators helped participants understand the underlying cause of their FF. This study highlighted the key roles of FLS staff in helping patients to recognize FF as a sign of underlying bone disease and encouraging adherence to care recommendations.

## 1. Introduction

Osteoporosis-related fracture, namely fragility fracture (FF), is defined as a fracture without trauma or that is caused by a fall from standing height or less [1,2]. Approximately one in three women and one in five men over 50 years of age will sustain at least one FF during their lifetime [3]. The risk of suffering a subsequent fracture doubles after fracture but is much higher immediately after fracture [4]. Despite the wide range of effective anti-osteoporosis medications, clinical practice guidelines and recommendations regarding bone-healthy lifestyles and fall prevention strategies [5], 80% of people who experience a FF do not receive any intervention or recommendations to prevent a subsequent FF [6,7,8,9,10,11,12]. To address the challenges of managing the health of patients who sustained a FF [13], Fracture Liaison Service (FLS) has been calculated to be a cost-effective post-fracture model of care. A few studies have shown that FLS facilitates evidence-based FF care and prevents subsequent fracture(s) [14,15,16]. FLS has up to five main objectives: (1) identify FF patients; (2) investigate their bone health status and risk of subsequent FF(s); (3) initiate medication to treat osteoporosis [5,17,18]; (4) make bone health recommendations [19]; and (5) integrate a patient-centered services management plan encompassing primary and secondary care and public health programs [20].

Patient perspective is increasingly recognized as a key component in redesigning healthcare processes and is advocated as a way to improve the healthcare of patients suffering from chronic diseases [21,22], like osteoporosis. Evaluation of healthcare services has begun to move beyond examining the provision of clinical care to consider the patient perspective as an essential component to enhance program performance [23]. Integrating patients’ perspectives into a health program evaluation ensures that it is rooted in their values and experiences [24]. While some authors evaluated the impact of FLS on adherence to health behaviors [25], none analyzed patient-related factors associated with adherence to recommendations made by such a service. Using a prospective design (with quantitative and qualitative data collection methods), this study aimed to identify factors associated with patients’ adherence at 12 months to various FLS recommendations including osteoporosis medication and bone health behaviors [26].

## 2. Materials and Methods

The experimental FLS Opti-Frac was launched in January 2013 in different health regions of the province of Quebec, Canada [27]. Patients were recruited between January 2013 and April 2015 and followed for 18 months until November 2016. Opti-Frac enrolled patients over 50 years of age, who had sustained a FF within three months of the recruitment date, were able to follow simple instructions, and had a primary care physician. Patients suffering from fractures of the skull, face, cervical spine, toe, hand, foot, ankle (according to prior fracture history and FRAX score [28]), and patella were excluded, as well as those presenting severe renal insufficiency (grade 4 or 5) or an advanced stage of cancer. Intervention participants received a telephone call from a trained FLS coordinator every three months and were provided with recommendations on (1) compliance with osteoporosis medication; (2) vitamin D supplementation; (3) practice of physical activity; and (4) participation in a public health fall prevention program. Recommendations were customized according to an algorithm based on FF practice guidelines and the participant’s adherence to bone health behaviors at the time of the call.

### 2.1. Quantitative Data

For this study, participants’ data were used to identify, through multivariate logistic regression models, the likelihood of participants’ adherence to FLS recommendations after 12 months of follow-up in the FLS (*n* = 354). The ethics committee of all the health regions involved approved the study (approval #MP-31-2013-497). All participants provided their informed consent before enrolling.

All variables included in the FLS database were self-reported by participants. Information gathered by coordinators included socioeconomic and clinical characteristics. FF sites were categorized as major FF (hip, vertebra, proximal humerus, and wrist), or minor FF (pelvis, ankle, radius, cubitus, tibia, fibula, ribs, clavicle, coccyx, and scapula) [29,30]. Participants sustaining multiple FFs during a single event were counted only once; major FFs took precedence over fractures at other sites [31]. Data related to the FLS coordinators’ professional background were obtained, i.e., nurses with a standardized order set for the initiation of osteoporosis treatment [32] or other disciplines without a similar prescription tool (biomedical specialist or physiotherapist). Data related to adherence to FLS recommendations were also extracted. Adherence to osteoporosis medication was defined as participants who persisted up to 12 months [33] and were compliant, i.e., took at least 80% of the medications [34,35,36,37]. Participants’ community pharmacists validated self-reported persistence and compliance data. If there was a discrepancy between the participant’s and the pharmacist’s information, the participant was considered non-adherent. Adherence to vitamin D supplementation was defined as taking at least 800 IU per day [5,38,39]. Doing at least 150 min of moderate- to vigorous-intensity physical activity per week was considered as adhering to the recommendation [40,41,42]. Vitamin D supplementation and participation in a fall prevention program were self-reported by participants, while a validated questionnaire, the *Community Health Activities Model Program for Seniors—CHAMPS*, was used to estimate practice of physical activity [41]. Fall prevention programs included any organized exercise program or fall prevention activities focusing on staying active and improving strength, balance, and mobility, on creating a safe home environment, or on adopting fall-prevention behaviors [42].

Descriptive analyses were conducted to characterize the sample. Missing data for adherence status were imputed using the last value carried forward, except for the practice of physical activity, which was collected only at 12 months. All independent variables were first correlated with adherence status to the four key recommendations through bivariate analysis, using chi-square or Fisher’s exact tests. Sub-group analyses by gender were also generated. All statistically significant variables at the 10% level in bivariate analyses were entered in multivariate logistic regression models. This interim step significance level was chosen to avoid missing important predictors of adherence. Logistic regression models were then fit to examine multivariate relationships between the outcomes of interest and the predictive variables and were repeated for men and women separately. The sample size was more than sufficient based on the number of variables assessed (including nominal variables and level of variable) [43]. Significance in the multivariate models was inferred at *p* < 0.05. Statistical analysis was performed with SPSS 24 (IBM Corp., Released 2015. IBM SPSS Statistics for MacIntosh, Version 24.0. Armonk, NY, USA). Table 1 summarizes the definitions and categorization of all variables. Logistic regression assumptions were tested to ensure valid results [44].

### 2.2. Qualitative Data

To provide a deeper understanding of the quantitative analysis findings, semi-structured in-depth interviews were conducted between March and July 2015 with a sample of 16 participants who had completed the 12-month assessment described above. Participants were selected based on purposeful sampling to enhance qualitative rigor [45]. An effort was made to select participants who were considered adherent to any one of the four FLS recommendations. Interviews were held at the participant’s preferred location and time in order to create an optimal environment for the discussion. Sixteen participants were identified across the three participating health regions for the qualitative phase of the study. Face-to-face interviews were conducted with eleven of the participants and the other five were interviewed by telephone. Data saturation was achieved after these interviews, i.e., when no new themes emerged. Open-ended interview questions focused on identifying facilitators and barriers to the participant’s adherence to each recommendation (Box 1). Interviews lasted approximately 40 to 50 min. All interviews were audiotaped and transcribed in full, and then carefully reviewed by the interviewer. NVivo version 10 (QSR International Pty Ltd. Version 10, 2012, Melbourne, Australia) was used to code and organize the transcribed data into themes. This allowed an iterative coding process that identified inconsistencies while facilitating updates of the coding system [46]. The coding process assigned sentences or paragraphs to a theme. Each theme was revisited and, if necessary, moved, merged, or divided into different sub-themes. Once the interviewer had coded the transcripts, a second analyst independently coded the material. Any differences were discussed until consensus was reached.

Box 1. **Key questions from the interview guide.**

1. What happened that caused the fracture?
2. What would have been different if your injury
had been sustained when you were younger?
3. What was the state of your bones when you
sustained your injury?
4. What made you decide to take/not take your
medication for your bones?
5. What made you decide to adopt/not adopt a
healthy lifestyle for your bones (vitamin D and physical activity)?
6. What made you decide to register/not register
for a fall prevention program?

## 3. Results

Quantitative variables used in the regression models are summarized in Table 1. Considering all the participants who completed at least 12 months of FLS follow-up (*n* = 354), the mean age was 66.6 (standard deviation 10.1) years. FF involved the wrist or forearm in 141 participants (40%), proximal humerus in 75 (21%), hip in 33 (9%), ankle in 29 (8%), vertebra in 11 (3%), and other sites in 65 (18%) participants. Among participants, 13 (4%) had FF at multiple sites. Table 1 presents the participants’ characteristics at baseline. Although all participants had underlying osteoporosis, only 105 of 354 (29.7%) were aware of their diagnosis upon recruitment into the FLS. Of note, most variables did not differ by sex, with the exception of income (significantly more men were in the higher income categories, *p* = 0.004) and awareness of one’s osteoporosis diagnosis (10.4% for men vs. 32.7% for women; *p* = 0.001). After 12 months of follow-up in the FLS, half the participants were considered adherent to osteoporosis medication (47.2%, 95% confidence interval (CI) 42.0–52.4), vitamin D supplementation was relatively high (76.6%, 95% CI 72.2–81.0), practice of physical activity was moderate (35.0%; 95% CI 30.0–40.0), and participation in fall prevention programs was reported by half the participants (52.5%, 95% CI 47.3–57.7). No statistical differences were observed in any of these percentages between men and women (data not shown). 

### 3.1. Correlates of Adherence to FLS Recommendations

Table 2 shows the variables entered in the regression models as potential predictors of participants’ adherence to FLS recommendations, as well as the corresponding adjusted odds ratios (ORs), 95% confidence intervals, and *p*-values.

### 3.2. Themes Emerging from Interviews

The profile of the participants interviewed is shown in Table 3. Whereas the quantitative results focused mainly on personal factors, the interviews allowed for the identification of the interpersonal dimension, which had an impact on participants’ adherence status. Three main themes emerged from the interviews: (1) FF as a sign of underlying bone disease; (2) support and feedback from the FLS; and (3) participants’ personal constraints.

#### 3.2.1. FF as a Sign of Underlying Bone Disease

The participants interviewed repeatedly reported that a clear understanding of their underlying bone disease was a key factor in convincing them of the importance of engaging in preventive behaviors. For various reasons, some participants had to wait until they enrolled in the FLS to find out that they had osteoporosis. The FLS coordinator provided this information by talking with the participants at regular intervals. Thus, participants enhanced their understanding of the cause underlying their FF, i.e., osteoporosis, as well as available treatments and lifestyle behaviors to prevent its progression. When participants had a good understanding of osteoporosis and its impact, they were more predisposed to recognize the relevance of the coordinator’s recommendations. Participants reported that this information should be known as soon as possible to help them manage their bone health as well as facilitate their adherence to FLS recommendations, such as osteoporosis medication. One participant who was adherent to all recommendations commented:

If I‘d known before, I would have started taking drugs against osteoporosis at that time and I wouldn’t have had a fracture. It would have really helped me.

(Participant 12, male, 65 years)

Information relating to the participants’ FF from different healthcare professionals across the healthcare continuum seemed to vary considerably. Inconsistency in the information received (and/or lack of understanding thereof) was one of the main barriers to adhering to FLS recommendations. Thirteen of the participants interviewed did not identify their FF as a sign of osteoporosis. Many health professionals seemed to have given incorrect or oversimplified explanations to participants about the state of their bones. For example, participants reported that some health professionals told them that their FF was healed, but the distinction between the underlying bone fragility and their FF healing was not clearly explained. On the other hand, some participants reported taking their osteoporosis medication although they did not know exactly why it had been prescribed. This misinformation appeared to interfere particularly with adherence to osteoporosis medication and participation in fall prevention programs.

#### 3.2.2. Support and Feedback from the FLS

Support and feedback from the FLS coordinator was appreciated as boosting adherence to recommendations. This support met the participants’ need to be monitored for their bone health after their FF. From their perspective, repeated telephone contacts and discussions with the coordinator strengthened their belief in the importance of improving their bone-healthy lifestyle. One participant who was compliant with vitamin D supplementation and had participated in a fall prevention program said:

Being in such a program confirmed that I was doing the right thing. The fact that they took care of me, they called me, they were serious, they encouraged me, and they congratulated me for what I was doing, I was grateful, and I didn’t give up!

(Participant 2, female, 55 years)

The impact of feedback from the FLS coordinator appeared to be greater when the participants’ trust in their family physician was high. Most participants mentioned trusting their family physician to act in their best interests; others said that their trust was mostly based on their physician’s medical competency. As part of the Opti-Frac program, participants’ family physicians were informed about their patients’ osteoporosis and FF by the FLS coordinator. Family physicians could then support their patients and discuss strategies to prevent subsequent FF(s), which could reinforce key messages delivered by the FLS coordinator. With respect to medication, some participants had great faith in their physician. To illustrate this, a participant who was compliant with all recommendations said:

My doctor recommended that I take it. I am obedient. When he asks me to take medication, I take it. I don’t argue with that.

(Participant 4, male, 79 years)

While support, trust, and respect were identified as essential elements for an optimal participant–healthcare professional relationship, which could in turn lead to better adherence, a loss of trust between participants and their physicians made it harder to persuade participants of the need for preventive behaviors. This situation had consequences for the participants as they became confused about the recommendations and felt the need to rely on other health professionals to better manage their osteoporosis. One participant explained:

My family doctor is no good. Most of the time, I have to see another doctor who prescribes medication for me. I don’t trust her with pills.

(Participant 10, female, 60 years)

#### 3.2.3. Participants’ Personal Constraints

Some of the participants interviewed mentioned that personal constraints limited their adherence to some FLS recommendations. More specifically, persistent musculoskeletal pain was mentioned as a common condition that had a negative impact on their practice of moderate-to-vigorous physical activities. Pain as the main cause of physical activity limitations was reported independently of poor health. In addition, misconceptions about the relationship between physical activity and pain seem to persist in the general population. One participant who did not do any physical activity at all explained that she had more difficulty doing her daily life activities compared to before her FF, so the fear of additional pain restricted the practice of physical activities:

Since I have a lot of pain in my knees, I give them a break and I don’t walk. I don’t do any exercise.

(Participant 14, female, 83 years)

Concerns about falls were identified as another barrier to doing physical activity. Fall-related fears often stemmed from the participant’s conviction of bone vulnerability and (re)fracture risks as a result of osteoporosis. The combination of (threatening) information conveyed by some health professionals and the experience of pain and discomfort strengthened this conviction. Participants who did not realize physical activity could help them reduce their risk of subsequent FF(s) had greater concerns about subsequent fall-related FF(s). Consequently, participants reported reducing their physical activity, in terms of both duration and intensity. One of the participants who did not do enough moderate-to-vigorous physical activity described her concerns about falling:

The [location of the] physical activity is far away. I can’t go there in the winter because I’m afraid of falling. I can’t afford to fall: I have osteoporosis problems.

(Participant 13, female, 64 years)

As illustrated in the above excerpt, some participants also mentioned barriers to accessing fall prevention programs, like cost and transportation issues. Notwithstanding those barriers and some other factors over which the participants had little control, such as their perceived health condition, education level, or age (as hinted by the regression models for women), participants noted that being followed in a FLS was linked to better access to healthcare. They argued that it helped them navigate the healthcare system. One participant who was economically disadvantaged and considered himself in poor health mentioned the help he received from the FLS coordinator to get access to adapted transportation so that he could participate in fall prevention programs. He said:

To be followed [by the FLS coordinator] ensured that I was not left alone because I didn’t know which door to knock on.

(Participant 11, male, 68 years)

## 4. Discussion

Patient-related factors that influence adherence to FLS recommendations identified in this study could inform further refinement of healthcare services and public health osteoporosis-related programs. Awareness of one’s diagnosis of osteoporosis was identified as an independent predictor of adherence to osteoporosis medication, adequate vitamin D supplementation, and suboptimal practice of moderate-to-vigorous physical activity. In-depth interviews also showed that the support and feedback provided by the FLS coordinator helped participants understand the underlying cause of their FF and was a major facilitator of participants’ adherence to FLS recommendations. On the other hand, participants’ personal constraints such as fear of falling or barriers to accessing fall prevention programs could impact negatively on patients’ adherence to bone health recommendations.

Fewer than one in three participants in the entire cohort (*n* = 354) knew that they had osteoporosis (either defined by densitometric measure or recent fracture) when they enrolled in the FLS. The quantitative results showed that inadequate information received (and/or lack of understanding thereof) by patients from some health professionals at the time of their FF could greatly impact the patients’ perception of the risk of subsequent osteoporosis-related FF(s). Thus, they are unlikely to understand their disease and the relevance of recommendations to prevent subsequent FF(s), as other studies have shown for recommendations related to medication [47,48] and vitamin D supplementation [49]. To optimize the delivery and effectiveness of post-FF interventions, FLS should engage in effective communication between all health professionals along the continuum of FF care, as well as in non-conflicting messages to participants. Such communication should focus on participants’ understanding of their diagnosis of osteoporosis and its impact.

Both quantitative and qualitative findings from this study suggested that support and feedback from a FLS coordinator facilitated participants’ adherence to bone health recommendations, regardless of their professional background and training. FLS coordinators could not only discuss osteoporosis and its impact with participants but also facilitate the relaying of information to participants’ family physicians to improve the continuity of care. By sharing such information, coordinators enabled family physicians to leverage their patients’ trust in them to enhance osteoporosis management. Participants’ trust in their physician was confirmed as a factor influencing medication adherence in other contexts [50,51]. Support and feedback from FLS coordinators were also shown to simplify access to organized fall prevention programs, since the Opti-Frac coordinators were trained to adapt their recommendations to the participants’ needs, preferences, and context.

Regression models fit for this study revealed that some personal factors influenced adherence to bone-healthy lifestyles. Among other factors, being aware of one’s osteoporosis diagnosis was an independent predictor for not doing enough moderate-to-vigorous physical activity. Qualitative findings deepened this information by showing that fear of falling and other personal constraints, such as pain or poor health, were viewed as reasons to engage in less vigorous physical activity. This study also suggested that being a younger senior (from 65 to 79 years old) could predict the practice of at least 150 min of moderate- to vigorous-intensity physical activity per week, which is consistent with the Study of Osteoporotic Fractures [52]. The fact that younger seniors are retired and have more free time could explain this association, as discussed by Baert et al. [53].

Systematic follow-up care through a FLS and regular communication between coordinators and participants provided a foundation to ensure a proper understanding of the relevance of fall prevention programs in the presence of osteoporosis. Of note, this study showed that high school as the highest level of education predicted non-participation in fall prevention programs while non-smoking predicted participation. Health professionals should pay special attention to participants with these characteristics. In addition, development by FLS coordinators of flexible strategies to accommodate participants’ needs, circumstances, and preferences should be considered in order to facilitate participation in fall prevention programs (e.g., exercise programs at a fitness center), as recommended by the National Institute for Health and Care Excellence guidelines regarding falls in older people [54,55]. 

This study had some limitations. First, the participants were all considered as a homogenous group of patients able to fully comply with all FLS recommendations (especially physical activity). It is probable, however, that some medical conditions may have prevented participants from participating in specific interventions. Second, all the study participants had a family physician. Therefore, results of this study may not be generalizable to participants who do not have a continuous relationship with a family physician, or who receive care from specialists only. A lack of statistical power might have precluded the identification of significant correlates in the sub-group analyses for sex. Finally, findings from the qualitative portion of this study may not be generalizable to the study sample or population [56]. However, thick descriptions of the phenomena observed should make the findings transferable, which is expected for qualitative study designs.

## 5. Conclusions

The aim of this study was to explore patient-related factors associated with adherence to FF care recommendations from a FLS. While the results of the quantitative analyses underlined the importance of personal factors such as age or recognizing one’s diagnosis of osteoporosis after a FF, the qualitative findings revealed that many interpersonal factors were also predominant, including the importance of health professionals’ communication and the FLS coordinator’s support. While the bond of trust between patient and physician also helps to better manage FF, a coordinated support system through a FLS coordinator could make a difference in enhancing participants’ engagement in their own care. The findings from this study can be used to adapt the way recommendations are provided in a FLS to help participants with FF improve their health.

## Figures and Tables

**Table 1:** Participants’ characteristics at baseline (*n* = 354). FF: fragility fracture.

	Variable; Definition (Reference Category)	Categorization	Total *n* (%)
**Socioeconomic characteristics**	Sex; sex of participant (male)	Male	48 (13.6)
		Female	306 (86.4)
	Age; age of participant at time of FF (80+)	50–64	170 (48.0)
		65–79	131 (37.0)
		80+	53 (15.0)
	Ethnic group; participant’s ethnic group (other)	Caucasian	338 (95.5)
		Other	16 (4.5)
	Education level; highest level of schooling completed by the participant (university)	Elementary school	39 (11.0)
		High school	136 (38.5)
		College	85 (24.1)
		University	93 (26.3)
	Income; participant’s income level (Can$60,000+)	<Can$20,000	129 (36.4)
		Can$20,000–Can$39,999	130 (36.7)
		Can$40,000–Can$59,999	43 (12.1)
		Can60,000+	34 (9.6)
		Did not want to answer	18 (5.1)
	Employment status; whether or not the participant had a job—“employed” indicated individuals working full-time, part-time, or occasionally (unemployed)	Employed	104 (29.4)
		Unemployed	250 (70.6)
	Living conditions; whether the participant lived with someone or alone (alone)	Living with someone	190 (53.7)
		Living alone	164 (46.3)
**Clinical** **characteristics**	Smoking; whether the participant was a current smoker or a non-smoker (smoker)	Smoker	53 (15.0)
		Non-smoker	301 (85.0)
	Site of FF; major FF included hip, vertebra, proximal humerus, wrist; minor FF included pelvis, clavicle, scapula, ribs, elbow, radius, cubitus, tibia, fibula, coccyx (minor)	Major	130 (36.9)
		Minor	222 (63.1)
	Osteoporosis diagnosis known; whether or not the participant reported diagnosis of osteoporosis (no)	Yes	105 (29.7)
		No	249 (70.3)
**Coordinator’s background**	Type of professional background; nurse or other—i.e., biomedical specialist or physiotherapist (other)	Nurse	74 (20.9)
		Other	280 (79.1)

**Table 2:** Correlates of adherence to Fracture Liaison Service (FLS) recommendations.

FLS Recommendations		Adjusted OR * (95% CI)	*p* **	Adjusted OR for Men * (95%CI)	Adjusted OR for Women * (95%CI)
**Adherence to osteoporosis medication (*n* = 354)**					
	Sex (female)	1.36 (0.69–2.65)	0.37	--	--
	Income (Can$60,000+)				
	Did not want to answer	1.16 (0.33–4.04)	0.79	1.28 (0.04–37.34)	1.38 (0.34–5.59)
	<Can$20,000	1.88 (0.74–4.79)	0.21	1.87 (0.17–20.22)	2.36 (0.79–7.00)
	Can$20,000–Can$39,999	1.31 (0.53–3.22)	0.59	0.42 (0.05–3.44)	1.78 (0.61–5.13)
	Can$40,000–Can$59,999	2.02 (0.75–5.41)	0.17	1.31 (0.12–14.82)	2.26 (0.71–7.13)
	Education level (university)				
	Elementary school	1.49 (0.62–3.53)	0.37	3.43 (0.15–79.85)	1.47 (0.58–3.73)
	High school	1.35 (0.71–2.55)	0.36	1.33 (0.19–9.14)	1.33 (0.66–2.67)
	College	1.50 (0.77–2.91)	0.24	6.56 (0.70–61.57)	1.29 (0.62–2.65)
	Site of FF (major)	1.38 (0.83–2.29)	0.21	2.37 (0.40–14.12)	1.36 (0.79–2.34)
	Osteoporosis diagnosis known by participant (yes)	2.47 (1.47–3.93)	**0.001**	0.51 (0.04–6.07)	**2.70 (1.62–4.52)**
	FLS coordinator’s background (nurse)	1.92 (1.07–3.46)	**0.03**	6.42 (1.01–40.68)	1.70 (0.90–3.24)
**Vitamin D supplementation (*n* = 354)**					
	Sex (female)	1.65 (0.82–3.32)	0.16	--	--
	Living with someone (yes)	0.63 (0.37–1.05)	0.08	0.43 (0.11–1.71)	0.69 (0.39–1.22)
	Osteoporosis diagnosis known by participant (yes)	2.34 (1.23–4.46)	**0.01**	0.70 (0.09–5.52)	2.71 (1.36–5.40)
	Income (Can$60,000+)				
	Did not want to answer	1.05 (0.26–4.23)	0.94	--	0.58 (0.12–2.89)
	<Can$20,000	0.86 (0.34–2.12)	0.74	2.26 (0.29–17.76)	0.60 (0.18–1.93)
	Can$20,000–Can$39,999	0.91 (0.38–2.23)	0.91	0.98 (0.21–4.60)	0.72 (0.22–2.34)
	Can$40,000–Can$59,999	2.40 (0.70–8.19)	0.16	4.97 (0.43–58.05)	1.55 (0.34–7.06)
**Physical activity practice (*n* = 301**)					
	Sex (female)	1.25 (0.59–2.64)	0.57	--	--
	Age (80+)				
	50–64	17.01 (3.85–75.19)	**<0.001**	--	**12.04 (2.64–54.81)**
	65–79	23.09 (5.20–102.49)	**<0.001**	--	**16.97 (3.74–77.00)**
	Education level (university)				
	Elementary school	0.84 (0.29–2.43)	0.74	2.14 (0.08–59.49)	0.71 (0.22–2.29)
	High school	0.88 (0.43–1.80)	0.72	3.60 (0.25–51.17)	0.77 (0.35–1.68)
	College	0.50 (0.24–1.03)	0.06	1.68 (0.14–20.74)	0.46 (0.21–1.01)
	Income (Can$60,000+)				
	Did not want to answer	0.32 (0.09–1.18)	0.09	--	**0.14 (0.03–0.65)**
	<Can$20,000	0.49 (0.17–1.38)	0.18	0.23 (0.01–5.83)	0.35 (0.10–1.17)
	Can$20,000–Can$39,999	0.44 (0.17–1.16)	0.1	0.63 (0.06–7.08)	**0.28 (0.09–0.92)**
	Can$40,000–Can$59,999	0.62 (0.21–1.80)	0.38	0.13 (0.01–3.22)	0.50 (0.14–1.73)
	Living with someone (yes)	1.51 (0.90–2.54)	0.12	0.52 (0.09–2.93)	**1.81 (1.02–3.20)**
	Osteoporosis diagnosis known by participant (yes)	0.45 (0.25–0.81)	**0.01**	--	**0.50 (0.27–0.92)**
**Fall prevention programs (*n* = 354)**					
	Sex (female)	1.59 (0.83–3.04)	0.16	--	--
	Age (80+)				
	50–64	0.74 (0.35–1.54)	0.41	1.57 (0.19–12.84)	0.56 (0.25–1.27)
	65–79	1.83 (0.94–3.58)	0.08	4.13 (0.56–30.31)	1.60 (0.77–3.33)
	Education level (university)				
	Elementary school	0.50 (0.22–1.13)	0.1	0.30 (0.02–4.31)	0.43 (0.18–1.05)
	High school	0.47 (0.27–0.84)	**0.01**	2.45 (0.59–10.18)	**0.32 (0.17–0.62)**
	College	0.65 (0.34–1.22)	0.18	1.00 (0.18–5.67)	0.54 (0.27–1.09)
	Employment status (employed)	0.65 (0.36–1.20)	0.17	1.47 (0.32–6.70)	1.45 (0.75–2.80)
	Smoking (non-smoker)	1.94 (1.02–3.67)	**0.04**	0.85 (0.14–5.40)	**2.16 (1.07–4.36)**

* All variables were included in the models; -- indicates a variable for which the small sample precludes estimation of the statistic; ** Bold values are significant with an alpha of 5%; OR = Odds ratio, CI = Confidence interval.

**Table 3:** Profile of the participants interviewed (*n* = 16).

Characteristic	Total *n* (%)
Female	10 (62.5)
Age (80+)	1 (6.3)
50–64	6 (37.5)
65–79	9 (56.3)
Education level (university)	3 (18.8)
Elementary school	2 (12.5)
High school	7 (43.8)
College	4 (25.0)
Smoker	2 (12.5)
Osteoporosis diagnosis known	5 (31.3)
Compliant with FLS recommendations	
Osteoporosis medication	7 (43.8)
Vitamin D supplementation	15 (93.8)
Practice of physical activity (at least 150 min)	6 (37.5)
Participation in fall prevention program	8 (50.0)
All recommendations	2 (12.5)
